# Novel Nanoarchitectures Based on Lignin Nanoparticles for Electrochemical Eco-Friendly Biosensing Development

**DOI:** 10.3390/nano11030718

**Published:** 2021-03-12

**Authors:** Cristina Tortolini, Eliana Capecchi, Federico Tasca, Riccardo Pofi, Mary Anna Venneri, Raffaele Saladino, Riccarda Antiochia

**Affiliations:** 1 Department of Chemistry and Drug Technologies, Sapienza University of Rome, P.le Aldo Moro 5, 00185 Rome, Italy; cristina.tortolini@gmail.com; 2 Department of Experimental Medicine, Sapienza University of Rome, Viale Regina Elena 324, 00166 Rome, Italy; riccardo.pofi@uniroma1.it (R.P.); maryanna.venneri@uniroma1.it (M.A.V.); 3 Department of Biological and Ecological Sciences, University of Tuscia, Via s. Camillo de Lellis snc, 01100 Viterbo, Italy; e.capecchi@unitus.it (E.C.); saladino@unitus.it (R.S.); 4 Departamento de Química de los Materiales, Facultad de Química y Biología, Universidad de Santiago de Chile, Casilla 40, Correo 33, Sucursal Matucana, Santiago 9170022, Chile; federico.tasca@usach.cl

**Keywords:** lignin nanoparticles, organosolv lignin, kraft lignin, electrochemical platform, glucose biosensor

## Abstract

Novel nanoarchitectures based on lignin nanoparticles (LNPs) were designed and realized for electrochemical eco-friendly biosensing development. Two types of lignin nanoparticles were utilized for the modification of a gold bare electrode, namely organosolv (OLNPs) and kraft lignin (KLNPs) nanoparticles, synthetized from a sulfur-free and a sulfur lignin, respectively. The electrochemical behavior of LNP-modified electrodes was studied using two electrochemical techniques, cyclic voltammetry (CV) and electrochemical impedance spectroscopy (EIS). Compared to the gold bare electrode, an evident decrease in the faradaic current and increase of the ΔE_p_ were observed in cyclic voltammograms. In addition, larger semicircles were registered in Nyquist plots. These results suggest a strong inhibition effect of the electron transfer reaction by LNPs layer, especially in the case of KLNPs. The modified electrodes, properly assembled with concanavalin A (ConA) and glucose oxidase (GOx), were successively tested as biosensing platforms for glucose, showing a sensitivity of (4.53 ± 0.467) and (13.74 ± 1.84) μA mM^−1^ cm^2^ for Au/SAMCys/OLNPs/ConA/GOx and Au/KLNPs/ConA/GOx biosensors, respectively. Finally, different layers of the KNLPs/ConA/GOx-modified Au electrode were tested, and the three-layered Au(KNLPs/ConA/GOx)_3_ showed the best analytical performance.

## 1. Introduction

In the last decade, significant effort has been devoted to the design and development of biosensors with improved characteristics in terms of selectivity, sensitivity, simplicity and response time. One of the most critical issues in biosensing design is the achievement of high sensitivity and selectivity together with an easy-to use format. To this aim, the choice of a proper biosensing interface is fundamental.

Several nanomaterials have been successfully used as electrochemical platforms for the immobilization of bioreceptor molecules with the desired orientation, allowing the retention of their biological activity. In addition to the immobilization step, the nanoplatforms perform as an analyte recognition site and a signal transduction and amplification probe for the enhancement of biosensor performances [1,2,3,4,5,6]. The nanoarchitecture in biosensor design is expected to offer high selectivity, accuracy and fast and real-time responsiveness, which are the main challenges in the current stage of biosensor development.

A variety of nanostructured materials have been utilized in the construction of biosensors, such as carbon-based materials like carbon nanotubes [7,8,9,10,11,12,13,14,15] and graphene [16,17,18,19,20,21], metal nanoparticles [22,23,24], quantum dots [25], nanowires [26,27] and magnetic nanoparticles [28]. Despite the extraordinary electrochemical properties of the nanobiosensors and their possibility of miniaturization for point-of-care (POC) use, drawbacks for clinical applications need to be solved. Among them, labeling and sample pretreatment steps require high technical experience for operation and preclude real-time analysis. Other limitations are associated with the biological affinity probes rather than in the actual electrical detection mechanism. Therefore, a better understanding of the immobilization chemistry and minimizing nonspecific binding are crucial issues in the development of biosensing devices.

**Scheme 1:**
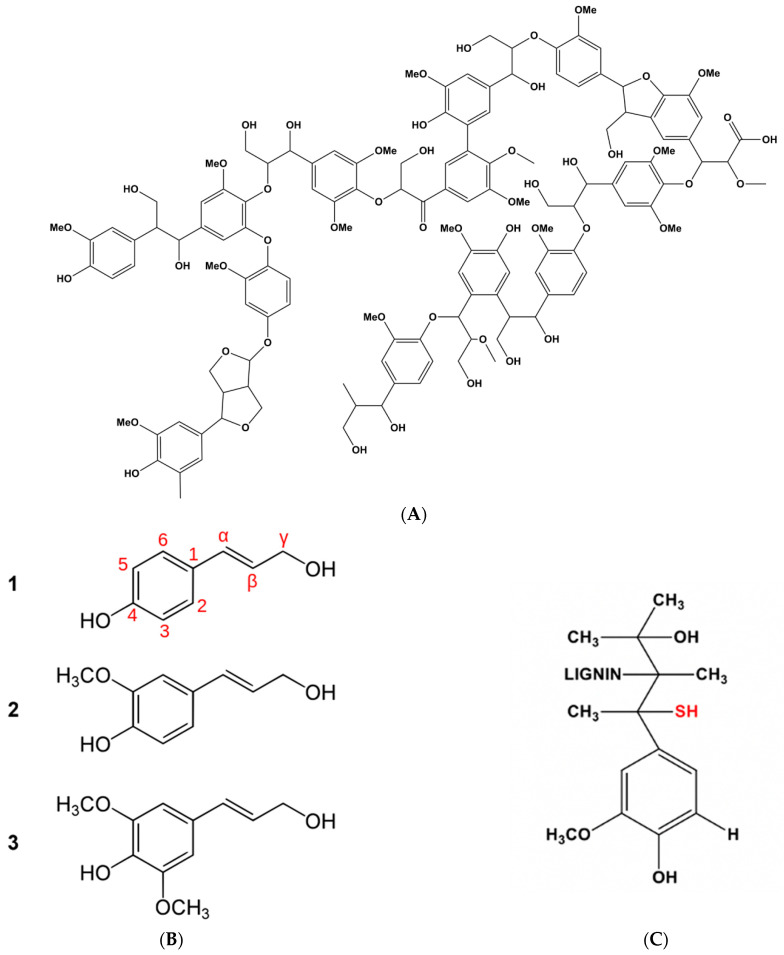
(**A**) Schematic structure of lignin; (**B**) the three common monolignols: paracoumaryl (**1**), coniferyl (**2**) and sinapyl (**3**) alcohol; (**C**) chemical structure of kraft lignin (KL).

In the last decade, lignin nanoparticles (LNPs) [29,30,31,32] have attracted a great deal of attention because of their biotechnological applications in different fields, including in antibacterial [33], antioxidant [34,35] and UV-adsorbent [36] agents, hybrid nanocomposites [37], drug delivery systems [38,39,40] and biocatalysis [41,42]. LNPs show novel characteristics such as a higher surface area, increased UV-adsorption capacity and radical scavenger activity compared to native counterparts. Lignin, the most abundant polyphenol in nature, is a polymer produced by the radical coupling of three phenylpropanoid units, as shown in Scheme 1 [43]. It is the only polymer in nature that contains such a large number of aromatic compounds, responsible for the beneficial properties associated with its aromatic character. Every year, approximately 50 million tons of lignin are produced worldwide as byproducts of the pulp and paper industry and biorefinery. Therefore, it is emerging as a new economic renewable raw material in a wide range of applications. During the lignin nanostructuration process, supramolecular π-interactions occur between the aromatic moieties, thus favoring electron transfer processes able to increase the electrochemical responsiveness. In particular, LNPs have been used as renewable and sustainable platforms for the immobilization of redox enzymes, such as laccase [44], tyrosinase [45] and the enzymatic cascade of horseradish peroxidase (HRP) and glucose oxidase (GOx) [46]. The benign effect of the ordered immobilization of the two enzymes on the surface of LNPs has been reported, focusing on the role of concanavalin A (Con A), a lectin able to interact with the carbohydrate component of both glycoproteins HRP and GOx [46], in the increase in the activity of the system [47,48].

There are two types of lignins based on different extraction processes: sulfur lignin, produced by chemical pulping processes, including kraft lignin (KL) and ligno-sulfonates (LSs), and sulfur-free lignin, including alkaline lignin and organosolv lignin (OL), produced by bioethanol production processes [49]. OL and KL differ in their elemental composition, molecular weight and purity, the main difference being the presence of sulfur in KL (Scheme 1). KL is soluble in water while OL is insoluble in water and soluble in organic solvents [50]. KL and OL nanoparticles have been used for innovative applications and advanced materials [51,52,53,54].

In this work, we describe and compare the use of organosolv lignin nanoparticles (OLNPs) and kraft lignin nanoparticles (KLNPs) as novel nanoarchitectures for the modification of a bare gold electrode for electrochemical biosensor development. In particular, we discuss the preparation and the electrochemical characterization of LNPs-modified platforms and, as a proof-of-concept, their application in the development of a glucose biosensor. The electrode was assembled by the layer-by-layer (LbL) procedure, and the electrochemical and biocatalytic electrode performance was evaluated depending on the number of deposited layers, as well as on the role played by Con A in the orientation of the GOx immobilization. To the best of our knowledge, no reports are present in literature on electrode platforms based on LNPs-assembled multilayer films composed by LNPs/ConA/GOx for glucose detection.

## 2. Experimental Methods

### 2.1. Reagents and Apparatus

Glucose oxidase from *Aspergillus niger* (EC 1.10.3.2, 160 kDa; catalytic activity according to producer specification is 100–250 U per mg) and Con A from jack beans (*Canavalia ensiformis*) type VI were purchased from Sigma-Aldrich, Buchs, Switzerland, and stored at −18 °C.

All chemicals used were analytical grade and used without any further purification: sodium monobasic phosphate (Na_2_HPO_4_), sodium dibasic phosphate (NaH_2_PO_4_), potassium chloride (KCl), potassium ferricyanide (III) (K_3_[Fe(CN)_6_]), potassium ferrocyanide (II) (K_4_[Fe(CN)_6_], exaammineruthenium(III) chloride ([Ru(NH_3_)_6_]Cl_3_), cysteamine (Cys), (N-(3-dimethylaminopropyl)-N′-ethylcarbodiimide hydrochloride, commercial grade (EDC) and N-hydroxysuccinimide, 98% (NHS), purchased from Sigma-Aldrich (Buchs, Switzerland). The complex [Os(2,2′-bipyridine)2(4-aminomethylpyridine)Cl]PF_6_ (OsCmplx) used as an electrochemical mediator was synthesized according to a previously published protocol [55].

Lignin nanoparticles from organosolv lignin (OL) or kraft lignin (KL) were prepared according to nanoprecipitation procedures described in our previous works [47]. Briefly, a solution of OL or KL (2.0 mg in 1.2 mL of THF/H_2_O 5:1 *v/v*) was added to Milli-Q water (in 3.8 mL) to obtain OLNPs and KLNPs.

All solutions were prepared in phosphate buffer 0.1 M, KCl 0.1 M, pH 7.4 (PBS buffer). A solution of 1.1 mM K_3_[Fe(CN)_6_ and 0.1 M KCl in water was used in cyclic voltammetric experiments for determination of electroactive area (A_e_) using the Randles–Ševcik equation. High-purity deionized water (resistance: 18.2 MΩ cm at 25 °C; TOC < 10 µg L^−1^) obtained from Millipore (Molsheim, France) was used throughout experiments.

Electrochemical measurements were performed in a 5 mL thermostated glass cell (model 6.1415.150, Metrohm, Herisau, Switzerland) with a conventional three-electrode configuration with an Ag/AgCl/KClsat (198 mV vs. NHE) as a reference electrode (cat. 6.0726.100, Metrohm, Herisau, Switzerland), a glassy carbon rod as a counter electrode (cat. 6.1248.040, Metrohm, Herisau, Switzerland) and a gold electrode (diameter 3 mm) as a working eletrode (Au, cat. 6.1204.320, Metrohm, Herisau, Switzerland). A gold-screen printed electrode (Au-SPE, 220BT Metrohm, Herisau, Switzerland, Aux: gold; Ref: silver, diameter 4 mm) was also used as a working electrode.

Electrochemical impedance (EIS) was utilized to characterize the different modifications of the Au electrode surface. EIS measurements were carried out at an equilibrium potential called the open circuit potential (OCP) without bias voltage in the frequency range of 0.1–10^3^ Hz using an ac signal of 10 mV amplitude at a formal potential of the redox probe (0.22 V vs. Ag/AgCl), using Autolab Potentiostat/Galvanostat (Eco Chemie, The Netherlands). EIS measurements were carried out using 20 mL of PBS buffer solution containing a mixture of 5 mM Fe(CN)_6_^3−^/ Fe(CN)_6_^4−^ as an electrochemical probe.

### 2.2. Preparation of Gold-Modified Electrodes

The gold electrode (Au) was polished using polish paper and alumina powders, 0.3 and 0.05 μm, and sonicated in clean water, followed by electrocycling in 0.5 M H_2_SO_4_ from −0.3 to +1.7 V vs. Ag/AgCl, for 20 scans at 0.3 Vs^−1^ and from −0.3 to +1.7 V vs. Ag/AgCl, for 2 scans at 0.1 Vs^−1^.

**Scheme 2:**
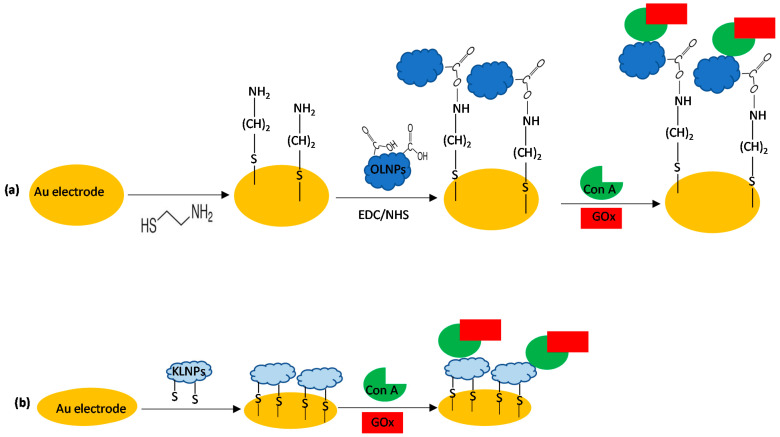
Preparation of gold modified electrodes: (**a**) Au/SAMCys/organosolv nanoparticles (OLNPs)/ConA/GOx and (**b**) Au/kraft lignin nanoparticles (KLNPs)/ConA/GOx.

The OLNP-modified biosensor was assembled by the following method: firstly, the cysteamine monolayer was prepared by soaking the clean gold electrodes (Au) in 18 mM cysteamine (CA) aqueous solution for 4 h at room temperature (RT) in darkness, washing the electrode thoroughly with water to remove physically adsorbed cysteamine. The carboxyl functions on the surface of organosolv lignin nanoparticles (OLNPs, 10 μL 2 mg mL^−1^) were activated with 20 μL of a mixture containing 0.5 mM EDC and 0.1 mM NHS in water for about 20 min. After removing EDC-NHS mixture and rinsing the surface of the electrode with PBS buffer solution, 20 μL of ConA (1 mg mL^−1^) was dropped on the working electrode. The immobilization of GOx was carried out by dropping 5 μL of a 5 mg mL^−1^ GOx solution (prepared in PBS buffer solution). Finally, the modified electrode (Au/SAMCys/OLNPs/GOx) was exhaustively rinsed with PBS buffer solution before electrochemical measurements (Scheme 2a).

The KLNPs biosensor (Au/KLNPs/GOx) was prepared by immersing the clean bare gold electrode in a solution of kraft lignin nanoparticles (KLNPs, 2 mg mL^−1^) for 2 h in darkness at room temperature. Then, ConA and GOx enzyme were added as described above for the first biosensor (Scheme 2b).

#### 2.2.1. Layer-by-Layer Nanoassembly

**Scheme 3:**
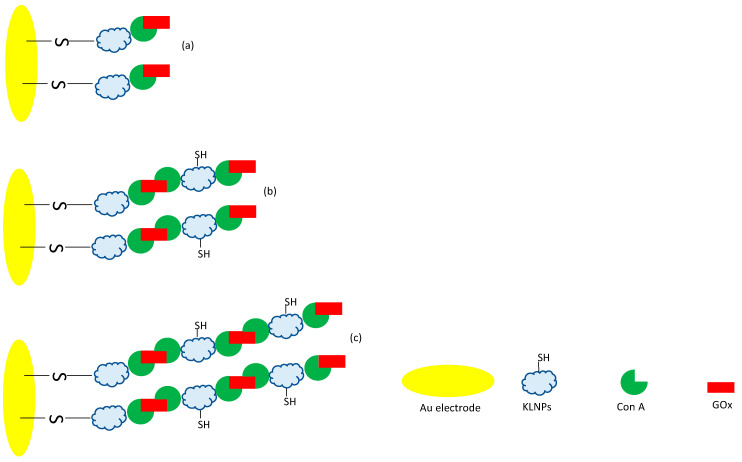
Schematic of the layer-by-layer (LbL) deposition process. Gold electrodes were successively functionalized with one (**a**), two (**b**) and three (**c**) layers of KLNPs, ConA and GOx by direct adsorption.

Two and three layers of the KNLPs/ConA/GOx biolayer were successively deposited onto the Au electrode according to a self-assembly LbL procedure (Scheme 3), as reported above for the first layer.

### 2.3. SEM Experiments

A high-resolution field emission scanning electron microscopy (SEM) (HR FESEM, Zeiss Auriga Microscopy, Jena, Germany) was used to investigate the morphology of the electrodes. All samples were prepared using gold plates (25 Å~ 25 Å~ 1 mm, ALS Co., Ltd., Tokyo, Japan) instead of gold electrodes.

## 3. Results and Discussion

### 3.1. SEM Characterization

**Figure 1:**
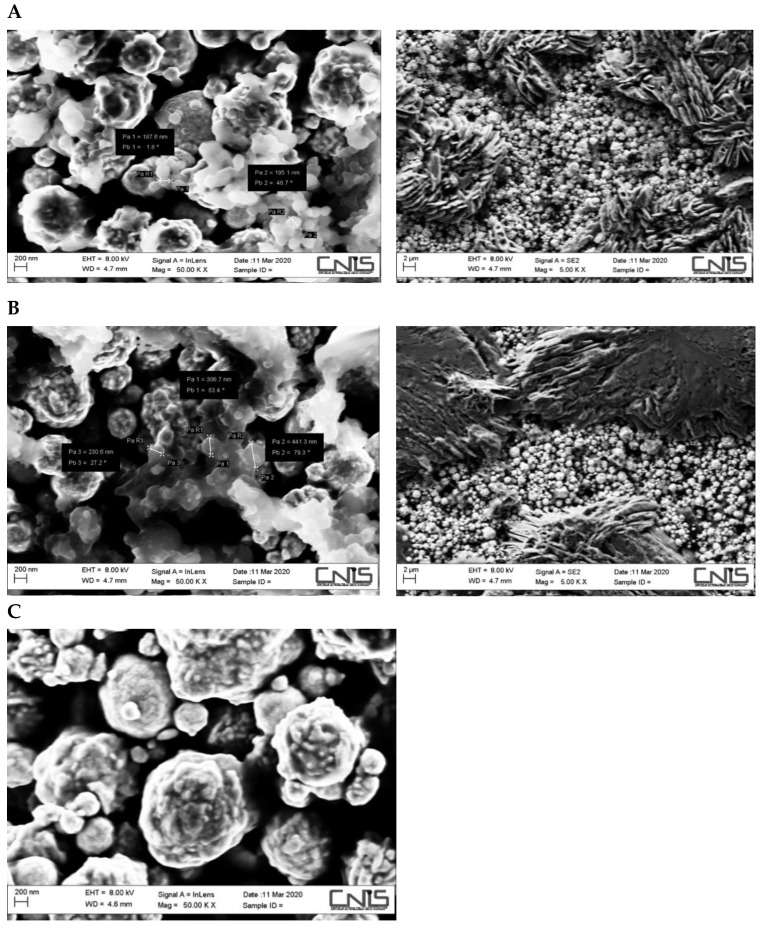
SEM images of OLNPs (**A**) and KLNPs (**B**) at two different magnifications and of Au bare electrode (**C**).

The surface morphology of OLNPs- and KLNPs-modified electrodes was studied using scanning electrode microscopy (SEM) experiments. Figure 1 shows the SEM images relative to the OLNPs (Panel A) and KNLPs (Panel B) Au-modified electrode, and a simple Au bare electrode (Panel C), as reference, at two different magnifications (50,000 and 5000× magnification). Regular spherical colloid particles with no significant aggregates or clusters for both types of lignin nanoparticles were observed. OLNPs result to be smaller than KLNPs with an average diameter between 150 and 200 nm, about half of that measured for KLNPs (average diameter 300–450 nm) (Figure 1, panel A and B, Mag 50 KX). In addition, KLNPs showed a better coverage of the gold electrode surface (Figure 1, panel A and B, Mag 5 KX), most likely because of the presence of the –SH groups able to directly bind the Au surface without the need of the SAM Cys spacer.

### 3.2. Electrochemical Characterization

The electrochemical behavior of the LNPs-modified electrodes was studied using two electrochemical techniques, cyclic voltammetry (CV) and electrochemical impedance spectroscopy (EIS), able to characterize the reversibility of the electron transfer process and the charge transfer resistance at the electrode–solution interface, respectively. The redox couple Fe(CN)_6_^3−/4−^ was used as an anionic electrochemical probe for examining the different platforms on a gold classical electrode [24].

#### 3.2.1. Cyclic Voltammetry Characterization

**Figure 2:**
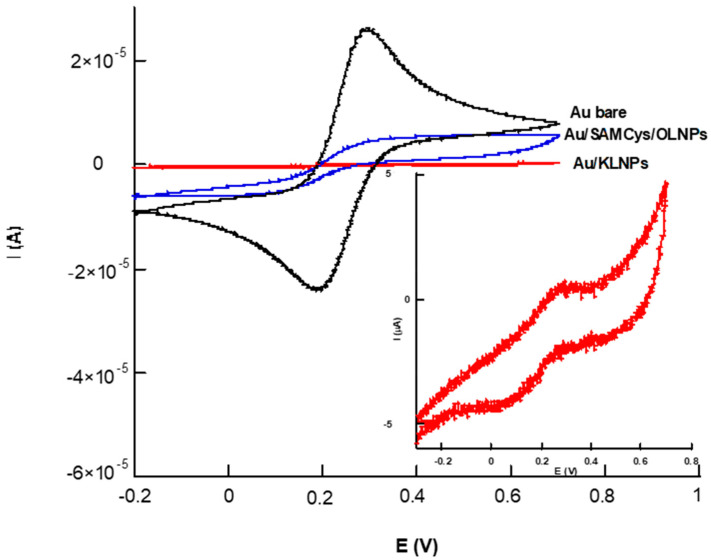
Cyclic voltammetry of 1.1 mM K_3_[Fe(CN)_6_ in PBS buffer on Au bare (black line), Au/SAMCys/OLNPs (blue line) and Au/KLNPs (red line), at ν = 100 mV s^−1^. In the inset, a magnification of the red curve.

Figure 2 shows the CV profile obtained before (black curve) and after the modification of a gold bare electrode with OLNPs (Au/SAMCys/OLNPs, blue curve) and KNLPs (Au/KLNPs, red curve) at a potential scan rate of 100 mV s^−1^.

KLNPs possess anchoring thiol groups in their structure, which allow the formation of direct S–Au bonds with the electrode. In the case of ONLPs, it was necessary to use a cysteamine self-assembled monolayer (SAMCys) to allow the connection to the surface of the gold electrode. Measurements of OLNPs directly adsorbed onto an Au electrode without the use of a SAM were previously carried out, and the voltammetric curves resulted to be stackable to the CVs obtained with the Au bare electrode (data not shown), because of the clearly visible leakage of the OLNPs in the cell solution during repeated scan cycles.

All voltammograms show a couple of redox peaks with a decrease in the peak current and an increase in the peak-to-peak distance after electrode modification with both ONLPs and KNLPs. The shape of the voltametric curve is similar for the three curves, indicating that the electron transfer (ET) process is under diffusion control. Further, the modification of the electrode surface leads to much lower peak current values and larger current separations between forward and reverse scan, suggesting a possible inhibition of the ET reaction caused by a “blocking behavior” exerted by the LNPs, which can enhance the insulating properties of the electrode without passivating its surface. This effect is more marked with KLNPs compared to SAMCys/OLNPs, most likely due to the formation of a more homogeneous KNLPs biolayer which almost completely covers the electrode surface. In the case of SAMcys/OLNPs, the presence of the spacer increases the distance between LNPs and the electrode surface, causing an increase in the electron tunneling, and at the same time, a decrease in the loading of LNPs on the electrode surface.

However, the presence of aromatic rings in the lignin molecules can somehow facilitate the ET due to the delocalized π-electrons, as confirmed by the two-peak shaped voltammograms (blue and red curves).

**Figure 3:**
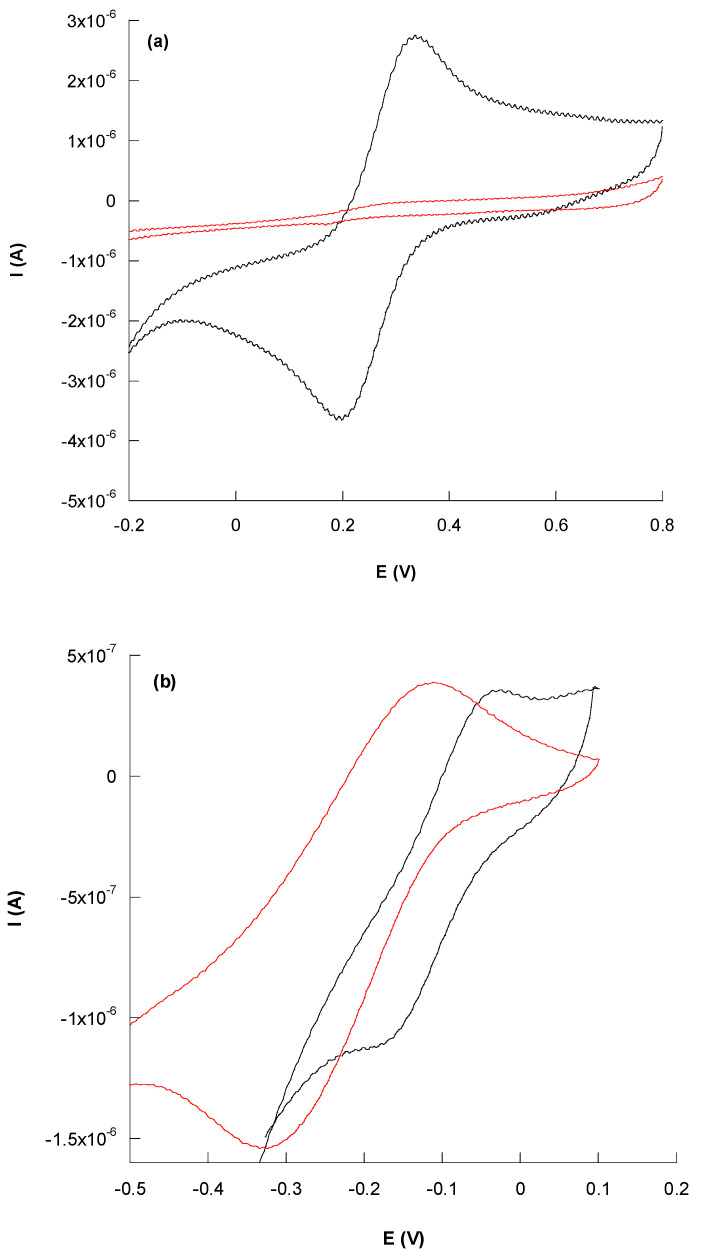
Cyclic voltammograms of 1.1 mM K_3_[Fe(CN)_6_ (**a**) and 1.1 mM [Ru(NH_3_)_6_]Cl_3_ (**b**) on Au (black line) and Au/KLNPs (red line). Experimental conditions: PBS buffer (pH 7.4), ν = 100 mV s^−1^.

The decrease in the faradaic current and the increase of the ΔE_p_ values can also be interpreted by the electrostatic repulsive interactions between the negatively charged Fe(CN)_6_^3−/4−^ and the LNPs, which show a negative Z potential value (−42 mV at pH 4.2) [56]. In order to better understand the extent of this effect, a comparison between the voltammetric responses of the commonly used negatively charged potassium ferricyanide and a positively charged redox probe, exaammineruthenium(III), was carried out and the results are reported in Figure 3. As it can be clearly observed, the negative effect in terms of peak currents and ΔE_p_ exerted by the KNLPs layer is present only with ferricyanide. When exammineruthenium(III) is used, the peak currents were almost unaffected by the lignin biolayer with a lower increase in the peak current separation. These results can be attributed to the anionic character of the lignin biolayer, which creates strong repulsive interactions with the negative ferricyanide, not present in the case of the cationic redox probe. Moreover, [Ru(NH_3_)_6_]^3+^ is known to be insensitive to surface modification within monolayer thickness as it is able to exchange electrons by tunneling [57].

Table 1 shows the electroactive area (A_e_, mm^2^), the heterogeneous electron transfer rate constant (*k*^0^, cm s^−1^) and the roughness factor (ρ) of the bare and the two modified electrodes. The A_e_ has been evaluated using the Randles–Ševcik equation [58], *k*^0^ was calculated using an extended method obtained by merging Klingler-Kochi and Nicholson-Shain methods, for totally irreversible and reversible systems, respectively [59], and the roughness factor (ρ) derives from the ratio of the electroactive (A_e_) to the geometric area (A_g_).

The Randles–Ševcik equation for a reversible process is as follows:

I_p_ = 2.686 × 10^5^ n^3/2^ A_e_ D_0_^1/2^ C_0_ v^1/2^ (1)

where I_p_ is the voltametric peak current (A), n is number of electrons, A_e_ is electroactive area (cm^2^), D_0_ is diffusion coefficient (7.6 × 10^−6^ cm^2^ s^−1^ for ferricyanide), C_0_ is the concentration (mol cm^−3^) and v is the scan rate (Vs^−1^). By using the slope of the plot of I_p_ vs. v^1/2^, the electroactive areas were calculated and the results are shown in Table 1. The A_e_ values of Au/SAMcys/OLNPs and Au/KLNPs resulted to be about 2- and 7-times smaller than that of the bare electrode, respectively. The coverage of the electrode surface with the non-conductive LNPs results in an obvious decrease of the A_e_ values, with a marked effect reported for the KNLPs, as explained above. Consequently, ρ values also decreased with electrode modifications because of the presence of the nanoparticle monolayer as the active surface (Table 1).

The heterogeneous electron transfer rate constant (*k*^0^) for the Au bare is much higher than those for the Au-modified sensors; however, the *k*_0_ value obtained with Au/SAMcys/OLPNs electrode is more than one order of magnitude higher than that obtained with KLNPs, most likely due to its particular nanoarchitecture realized by the SAM molecules, which allow an electron-tunneling effect through the layer.

#### 3.2.2. Electrochemical Impedance Spectroscopy Characterization

**Figure 4:**
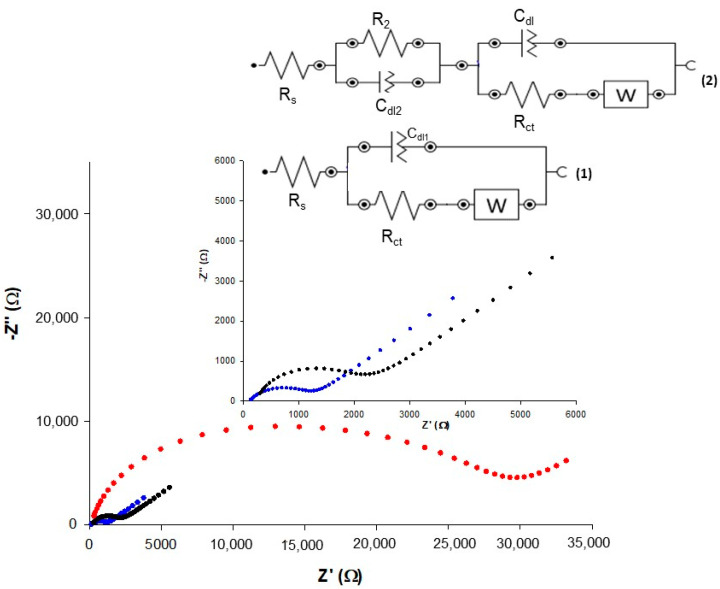
EIS response (Nyquist plots) of Au bare (blue curve), Au/SAMCys/OLNPs (black curve) and Au/KLNPs (red curve). The inset shows a magnification of blue and black curves and equivalent circuits used for fitting the experimental data, where (**1**) was used for Au bare and (**2**) for both modified electrodes. Experimental conditions: 5 mM Fe(CN)_6_^3−^/Fe(CN)_6_^4−^ in PBS buffer (pH = 7.4) at 10^3^ Hz.

EIS is one of the very sensitive techniques used for characterization of surfaces and for detecting biomolecular interactions occurring at the surface of an electrode [25,26,27]. Figure 4 shows the Nyquist plots for the bare and the two modified Au electrodes in the presence of 5 mM Fe(CN)_6_^3−^/^4−^ redox couple. The impedance measurements were carried out at formal potential of 0.22 V vs. Ag/AgCl.

The bare Au electrode exhibits a very small semicircle at a high frequency region and a low frequency straight line, indicating a reversible and diffusion-controlled ET process of the redox probe (Figure 4, blue curve).

On the contrary, lignin nanoparticle modified electrodes exhibit much larger semicircle formations because of the inhibition of the ET by the formation of the nano–lignin biolayer. In particular, it can be seen from Figure 4 that the SAMCys/OSLNP-modified electrode shows the formation of a semicircle in the high frequency region and a small straight line in the low frequency region (black curve), highlighting a quasi-reversible behavior of the redox probe and confirming a moderate blocking capacity of the biolayer. In the case of KNPLs, a very large semicircle over the entire range of frequencies used is observed, attesting a stronger blocking behavior and a high inhibition of the redox reaction.

The impedance data were simulated by different equivalent circuits, as shown in the Figure 4 inset. A Randles circuit was successfully applied to fit the Au bare electrode (Figure 4, inset (a)). The obtained simulation shows low resistance of charge transfer (R_ct_ = 725 ± 1 Ω cm^2^) suggesting a fast charge transfer between the redox probe and electrode.

After SAM formation and the amino-amidic bonding with OLNPs, the layer became thicker and the reaction slowed, and another equivalent circuit was proposed (Figure 4, inset b). The R_ct_ value increased to 1.83 ± 0.03 kΩ cm^2^, confirming the occurred modification by blocking of the interfacial charge transfer. Finally, the equivalent circuit fitting procedure applied to the impedance data of KNLP-modified electrode showed a very large R_ct_ value, as expected (R_ct_ = 15.4 ± 0.5 kΩ cm^2^).

These results are in good agreement with the results obtained by cyclic voltammetry discussed in the previous paragraph.

### 3.3. Catalytic Behavior of Au/SAMCys/OLNPs/Con A/GOx and Au/KLNPs/Con A/GOx Platforms

**Figure 5:**
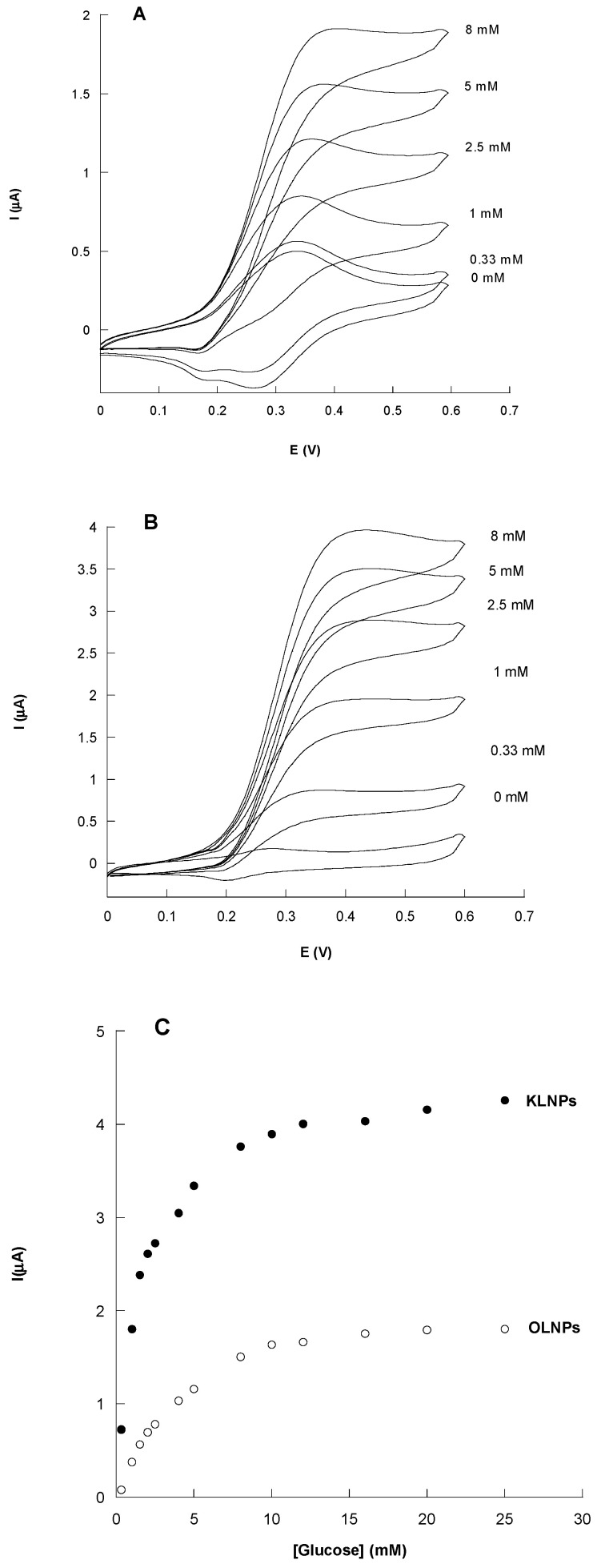
Cyclic voltammograms of a solution containing 0.1 mM OsCmplx at Au/SAMCys/OLNPs/Con A/GOx (**A**) and Au/KLNPs/Con A/GOx (**B**) in the absence and presence of glucose at different concentrations (0–8 mM). (**C**) Calibration plots of the glucose biosensors. Experimental conditions: N_2_-saturated in PBS buffer solution, pH 7.4, ν = 10 mVs^−1^, T = 298 K.

The glycoenzyme GOx, a well-studied system, was chosen as a model enzyme for studying the bioelectrocatalytic features of the two modified LNP electrodes, assembled with Con A as biosensing platforms. In order to confirm the enzymatic activity of the immobilized GOx, the electrochemical re-oxidation of an osmium complex (OsCmplx) was followed in the mediated oxidation of the FADH_2_ group of GOx and the subsequent oxidation of glucose. The Os-redox polymer was chosen because of its cationic character, which does not create negative repulsions with the negative lignin biolayer, showing a similar voltammogram (curve not shown) to that obtained in the case of [Ru(NH_3_)_6_]Cl_3_ (Figure 3, curve b), and at the same time because it is suitable to be used with the function of both mediator and support for many different redox enzymes [60]. Typical voltammograms (Figure 5, Panels A and B) demonstrated the catalytic activity of GOx assembled by Con A on LNP-modified electrodes, using OsCmplx as a redox mediator. The presence of Con A on the electrode increased the anodic current with a concomitant disappearance of the cathodic peak (S-shape), according to an electrocatalytic process. The resulting calibration plots are shown in Figure 5C (Panel C).

The kinetic and analytical parameters of the two proposed platforms are summarized in Table 2. A linear relation between the current response of the electrode and the amount of glucose in the concentration range 0.33–2.5 and 0.15–2.5 mM was obtained for Au/SAMCys/OLNPs/Con A/GOx and Au/KLNPs-SH/Con A/GOx biosensors, respectively. At higher concentrations, the amperometric response is no longer linear due to the saturation of the active site of the enzyme. The detection limit of the biosensors were found to be 0.11 and 0.05 mM, respectively, calculated using the relation 3σ/S, where σ is the absolute standard deviation of the intercept and S is the slope of the calibration curve [61].

The sensitivity resulted to be three times higher with KNLPs than OLNPs. This result, apparently in contrast with data previously reported, can be explained by taking into account the larger electrode coating of the KNLPs layer, which allows an immobilization of a much larger amount of enzyme. Moreover, the presence of Con A favors a correct orientation of the GOx enzyme during the immobilization process [47], enhancing the electrocatalytic performance of the system.

The apparent Michaelis–Menten constants, obtained by fitting the calibration curves, show a reduction of the K_M_^app^ value with KNLPs, suggesting a larger enzymatic affinity for glucose when KNLPs are used.

The best electrode platform was Au/KLNPs/Con A/GOx, and therefore this platform was used for further experiments.

### 3.4. LbL Functionalization of the Au/KLNPs/Con A/GOx Platform

**Figure 6:**
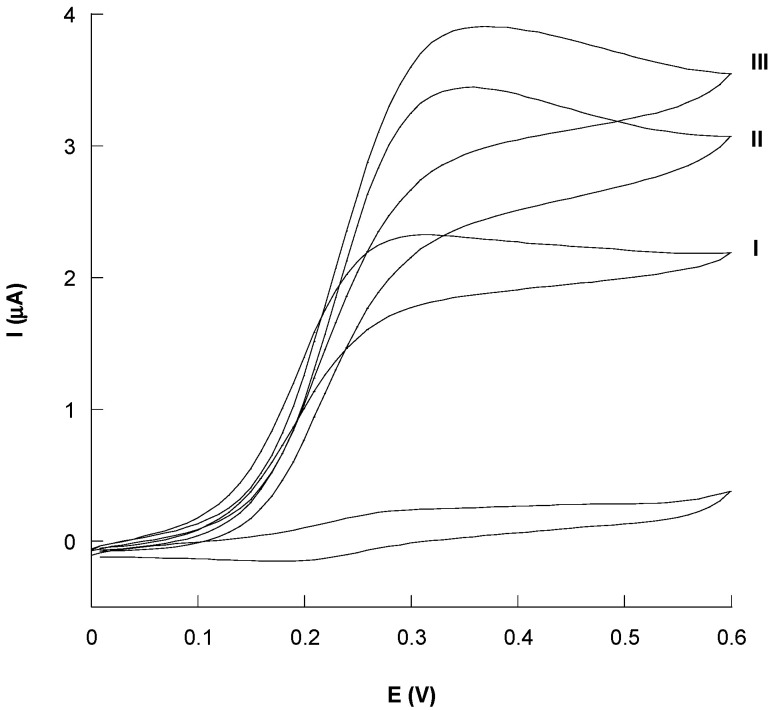
Voltammetric response of one (I), two (II) and three (III) biolayers of KLNPs/ConA/GOx on the Au-modified electrode in the presence of 2 mM glucose and 0.1 mM OsCmplx. Experimental conditions: PBS buffer (pH 7.4), T = 298, ν = 10 mV s^−1^.

Different layers of the KNLPs/CoA/GOx were assembled by repeating the steps described for the preparation of the Au-modified electrode (Section 2.2.1). The properties of the resulting glucose biosensor were measured by cyclic voltammetry. The amperometric current uniformly increases at increasing number of layers from one to three (Figure 6), reaching a saturation after the third layer (data not shown).

Comparing the voltammograms of the three-layers system, a two-times increase in the current signal was observed between the first and the third layer. Thus, the assembling of the three layers exhibited a remarkable improvement on the catalytic properties in the recognition of glucose, confirming that the LbL assembly process is able to enhance the performances of the biosensors in terms of sensitivity, reproducibility and stability [62,63]. In particular, the three-layered Au(KNLPs/CoA/GOx)_3_ biosensor showed an enhanced sensitivity of 15.85 µA/mM cm^2^, a linear range between 0.1 and 2.5 mM and a lower LOD of 0.02 mM, compared to the one-layered same platform. In addition, the increase in the current value clearly demonstrates the important role played by Con A, which allows a well-oriented immobilization of GOx and by LNPs due to their boosting effect in the activity of the overall electrocatalytic system, as previously hypothesized in our previous work [47].

## 4. Conclusions

In this work, a study on the use of organosolv and kraft lignin nanoparticles for the design and development of novel nanoassembled electrochemical platforms is presented. The data reported that the properties of the nanoparticles are governed by the type of lignin used. A blocking behavior was shown by both LNP-gold-modified electrodes because of the insulating nature of the LNPs. This effect is more pronounced with KLNPs compared to OLNPs, most likely because of the formation of a thicker KNLPs layer, which allows a better coating of the electrode surface. In addition, OLNPs were anchored to the Au electrode surface through a SAM molecule, which increases the distance between the LNPs and the electrode surface, with a consequent lower loading of the nonconductive LNPs and enhancement of the electron-tunneling effect through the layer.

The Au/KLNPs/Con A/GOx platform showed better electrocatalytic results compared to Au/SAMCys/OLNPs/Con A/GOx due to the efficient coating of KNLPs, which allows an immobilization of a larger amount of enzyme. Moreover, the presence of the lectin Con A favored the correct orientation of the GOx enzyme during the immobilization process, contributing to the enhancement of the electrocatalytic performance of the system. A self-assembly-driven modification strategy consisting of immobilizing three layers of KNLPs/CoA/GOx allowed the construction of a nanostructured film with the maximum catalytic current. This result confirms that LbL technique is a powerful tool to create functional nanocoatings with enhanced properties. These findings hold great application prospects for future applications of lignin nanoparticles in eco-friendly biosensor developments.

## Figures and Tables

**Table 1:** Comparison of electrochemical parameters before and after the modification of Au electrode: electroactive area (A_e_, mm^2^), electron transfer rate constant (*k*^0^, cm s^−1^) and roughness factor (ρ). Geometric area (A_g_, mm^2^) = 7.06.

Electrochemical Platform	A_e_ (mm^2^)	*k*^0^ (10^−3^ cm s^−1^)	ρ
Au bare	5.3	3.97 ± 0.79	0.75
Au/SAMcys/OLNPs	2.6	1.96 ± 0.34	0.37
Au/KLNPs	0.8	0.16 ± 0.36	0.11

**Table 2:** Electroanalytical and kinetic parameters of the glucose biosensors employing the proposed electrochemical platforms.

Electrochemical Platform	Imax (µA)	K_M_^app^ (mM)	Linear Range (mM)	Sensitivity (µA/mM cm^2^)	LOD (mM)	R^2^
Au/SAMCys/OLNPs/Con A/GOx	2.43 ± 0.11	2.30 ± 0.42	0.33–2.5	4.53 ± 0.467	0.11	0.968
Au/KLNPs/Con A/GOx	4.58 ± 0.04	1.37 ± 0.06	0.15–2.5	13.74 ± 1.84	0.05	0.947
